# Supplementary material for: Sensorimotor confidence during explicit motor adaptation
Source: bioRxiv. 2025 Nov 1:2025.10.31.685783. Preprint. [Version 1] doi: 10.1101/2025.10.31.685783 (PMC12636410; doi:10.1101/2025.10.31.685783)
Supplement: Supplement 1 [file NIHPP2025.10.31.685783v1-supplement-1.pdf]

bioRxiv preprint doi: https://doi.org/10.1101/2025.10.31.685783; this version posted November 1, 2025. The copyright holder for this preprint (which was not certified by peer review) is the author/funder, who has granted bioRxiv a license to display the preprint in perpetuity. It is made available under aCC-BY 4.0 International license.

Supplemental Materials

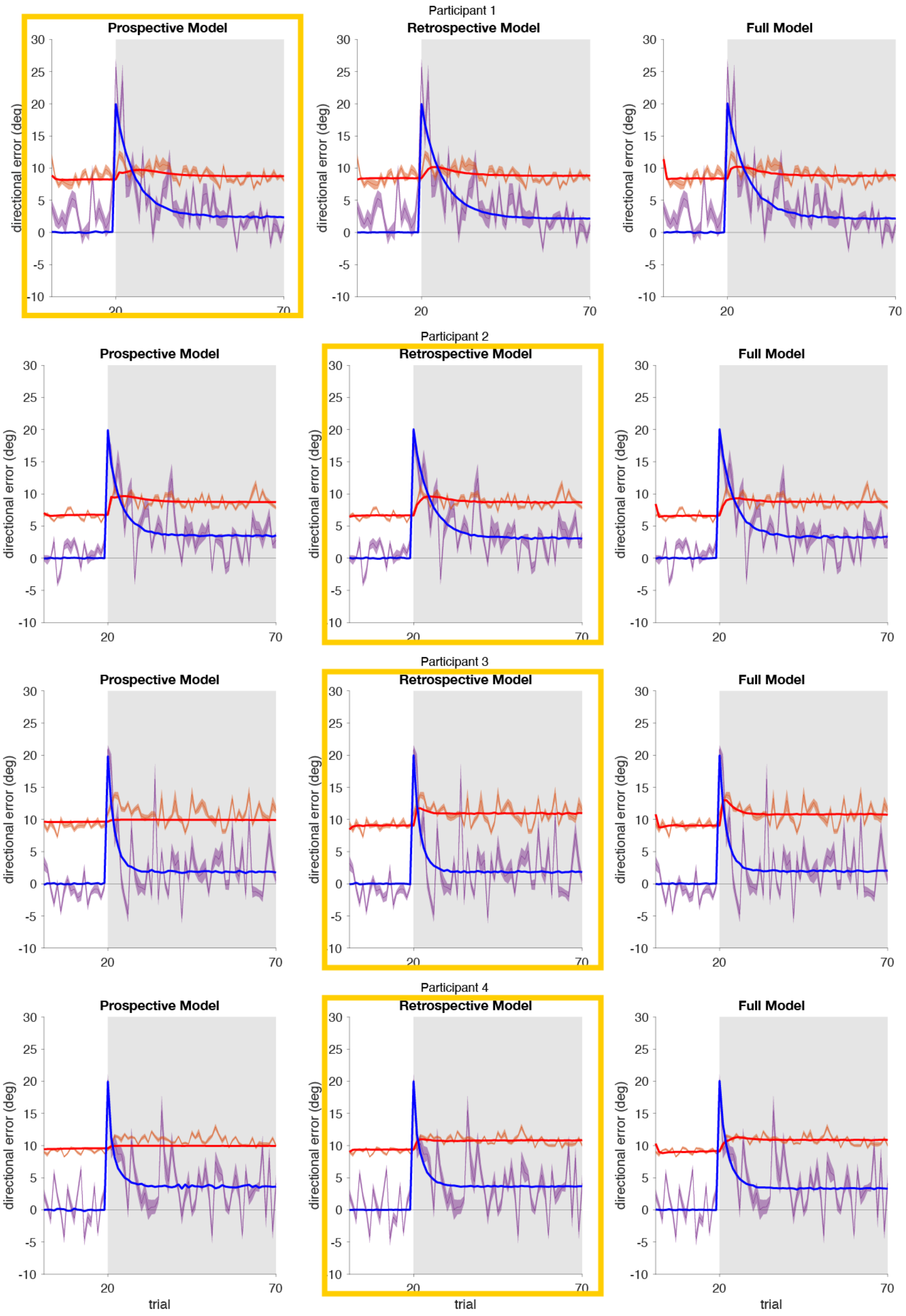

bioRxiv preprint doi: https://doi.org/10.1101/2025.10.31.685783; this version posted November 1, 2025. The copyright holder for this preprint (which was not certified by peer review) is the author/funder, who has granted bioRxiv a license to display the preprint in perpetuity. It is made available under aCC-BY 4.0 International license.

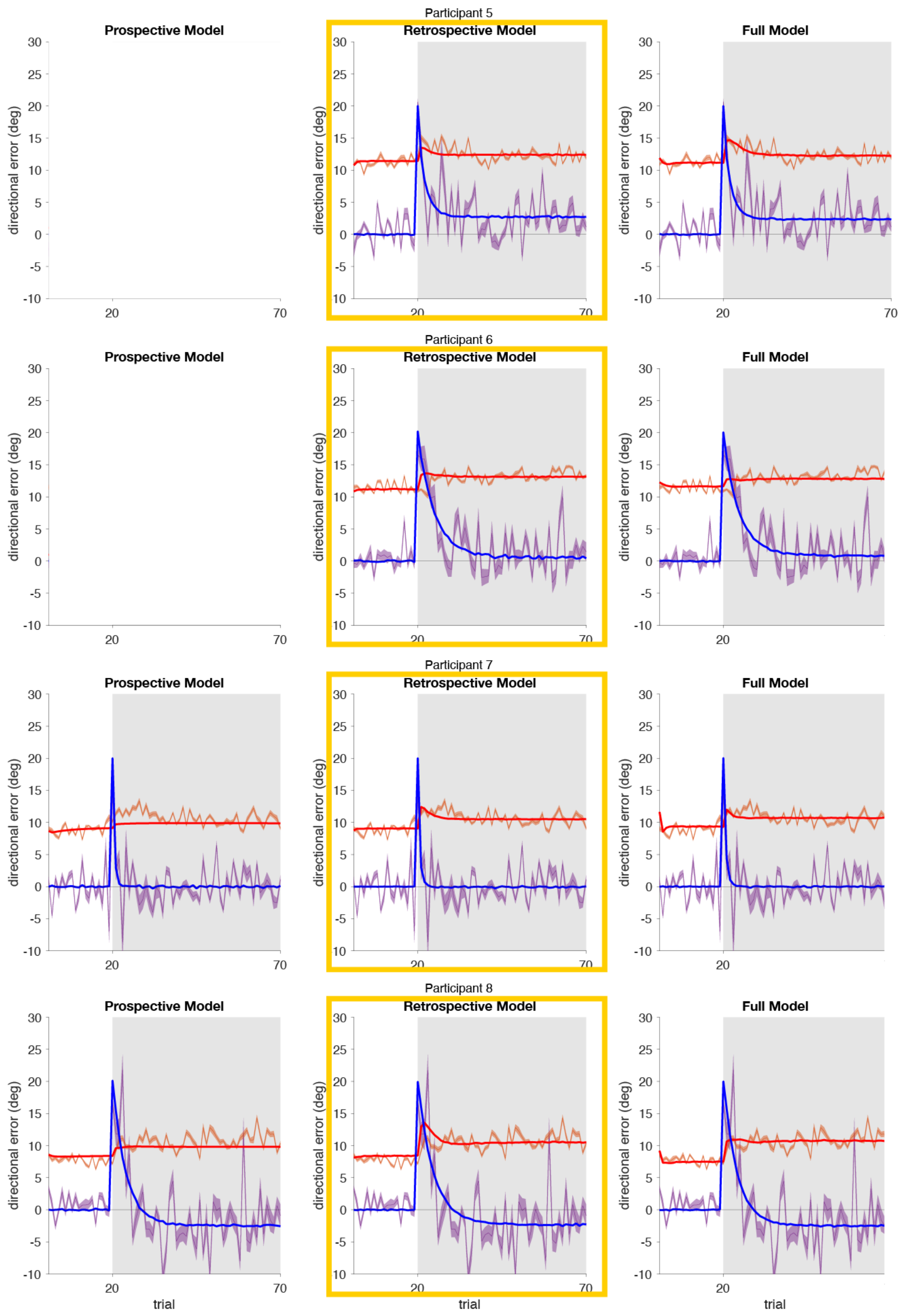

bioRxiv preprint doi: https://doi.org/10.1101/2025.10.31.685783; this version posted November 1, 2025. The copyright holder for this preprint (which was not certified by peer review) is the author/funder, who has granted bioRxiv a license to display the preprint in perpetuity. It is made available under aCC-BY 4.0 International license.

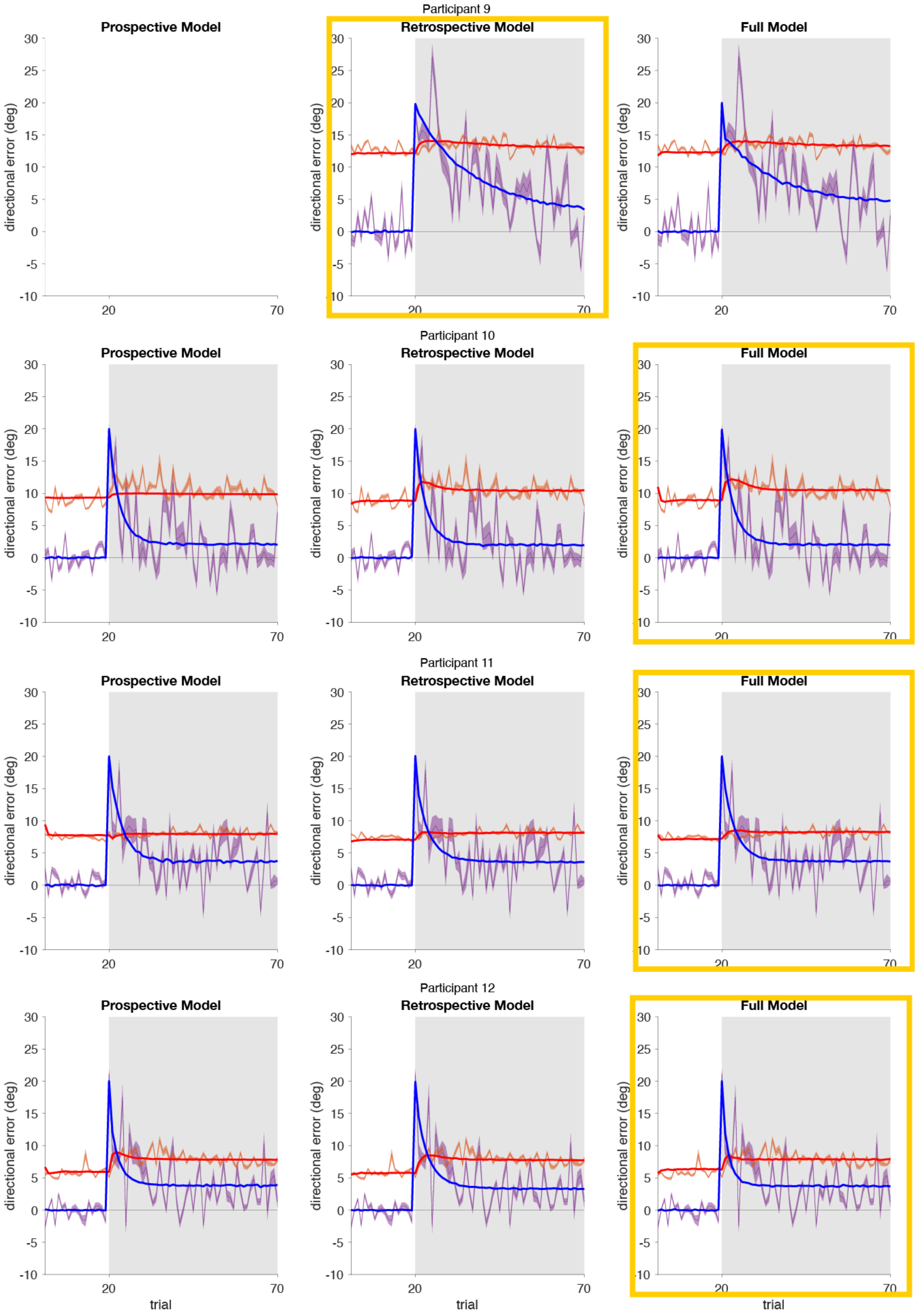

bioRxiv preprint doi: https://doi.org/10.1101/2025.10.31.685783; this version posted November 1, 2025. The copyright holder for this preprint (which was not certified by peer review) is the author/funder, who has granted bioRxiv a license to display the preprint in perpetuity. It is made available under aCC-BY 4.0 International license.

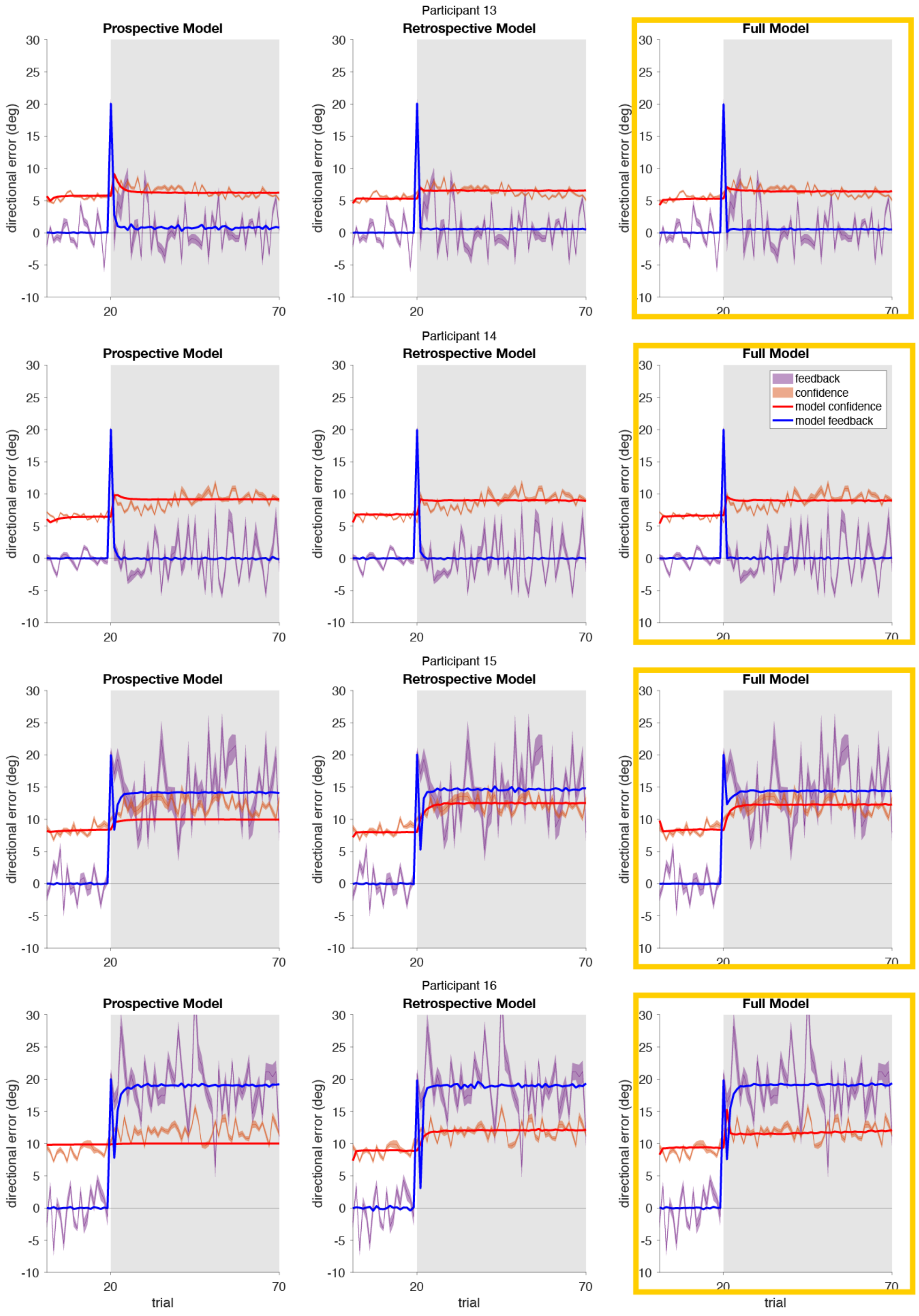

bioRxiv preprint doi: https://doi.org/10.1101/2025.10.31.685783; this version posted November 1, 2025. The copyright holder for this preprint (which was not certified by peer review) is the author/funder, who has granted bioRxiv a license to display the preprint in perpetuity. It is made available under aCC-BY 4.0 International license.

Supplement 1: Simulations of the models using the best-fit parameters compared to the behavioral data.
For each participant the simulated reach error (blue) and confidence report (averaged over 1000 stochastic simulations and 12 iterations, red) using the best-fit parameters for each model is overlaid on the behavioral data for that participant (reach error in purple, confidence in orange). The winning model for that participant, determined by cross-validated least squares, is highlighted with a yellow box. If a particular model never was the best fit for a given participant it has been left blank in the plots.

bioRxiv preprint doi: https://doi.org/10.1101/2025.10.31.685783; this version posted November 1, 2025. The copyright holder for this preprint (which was not certified by peer review) is the author/funder, who has granted bioRxiv a license to display the preprint in perpetuity. It is made available under aCC-BY 4.0 International license.

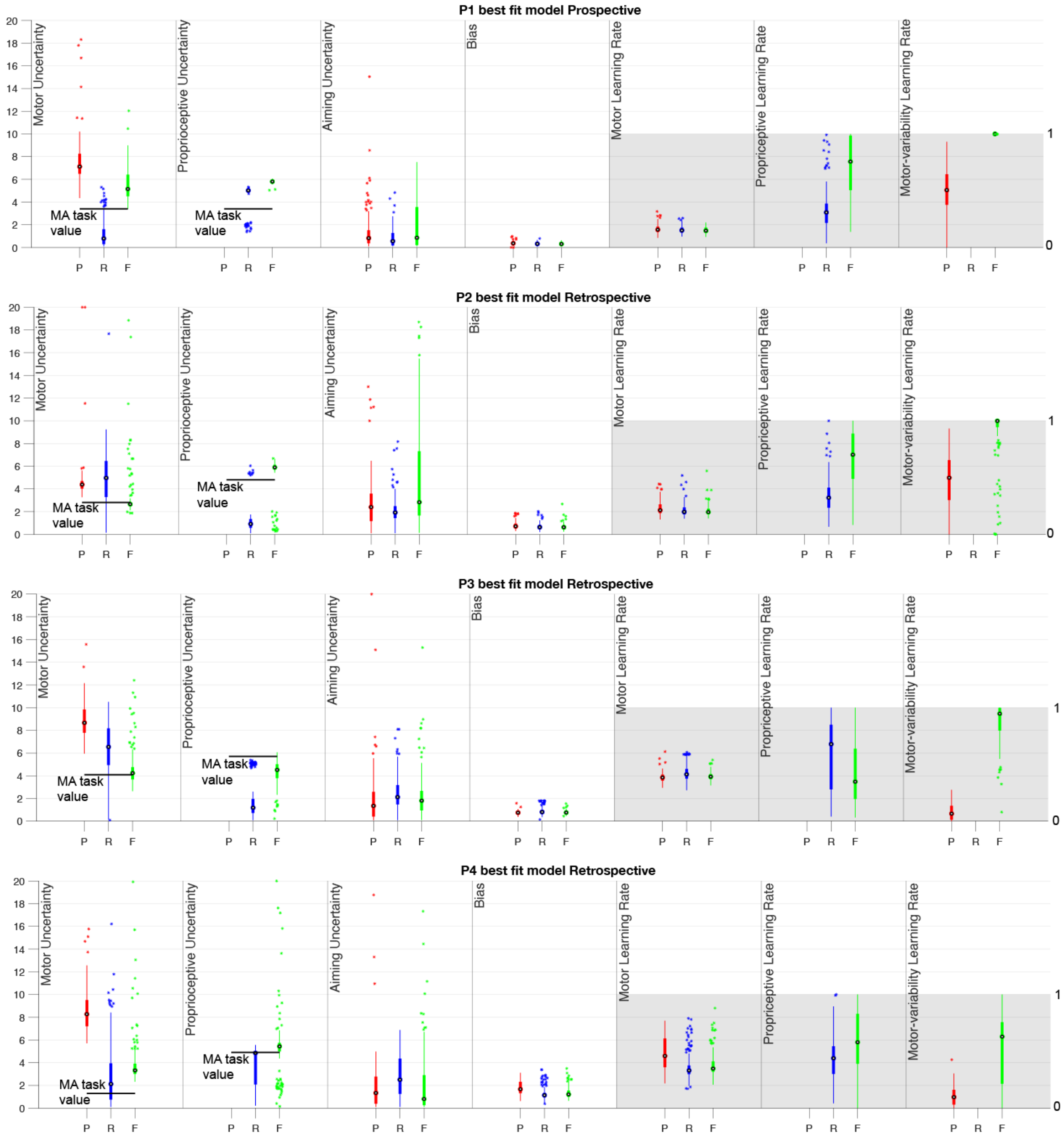

bioRxiv preprint doi: https://doi.org/10.1101/2025.10.31.685783; this version posted November 1, 2025. The copyright holder for this preprint (which was not certified by peer review) is the author/funder, who has granted bioRxiv a license to display the preprint in perpetuity. It is made available under aCC-BY 4.0 International license.

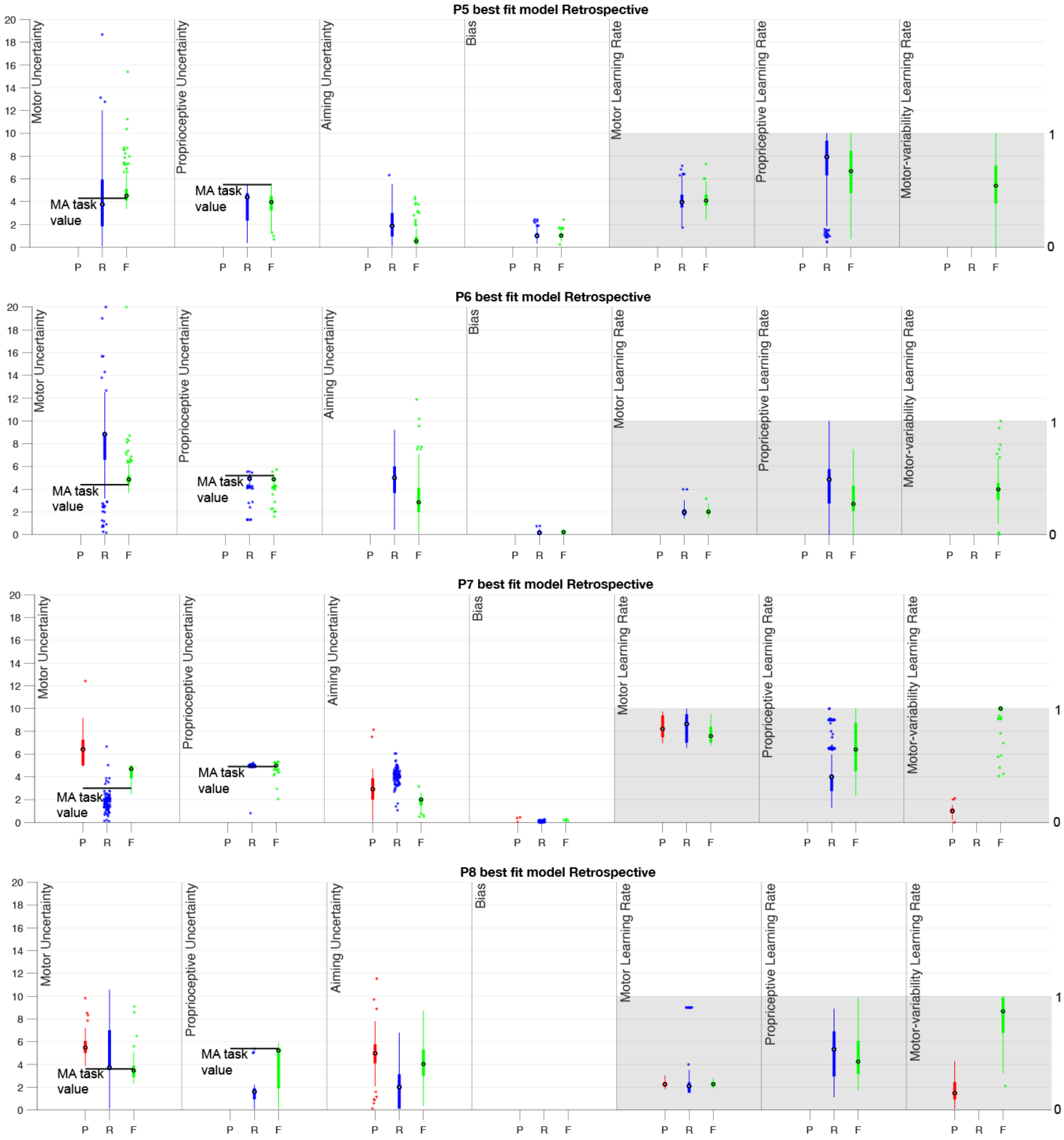

bioRxiv preprint doi: https://doi.org/10.1101/2025.10.31.685783; this version posted November 1, 2025. The copyright holder for this preprint (which was not certified by peer review) is the author/funder, who has granted bioRxiv a license to display the preprint in perpetuity. It is made available under aCC-BY 4.0 International license.

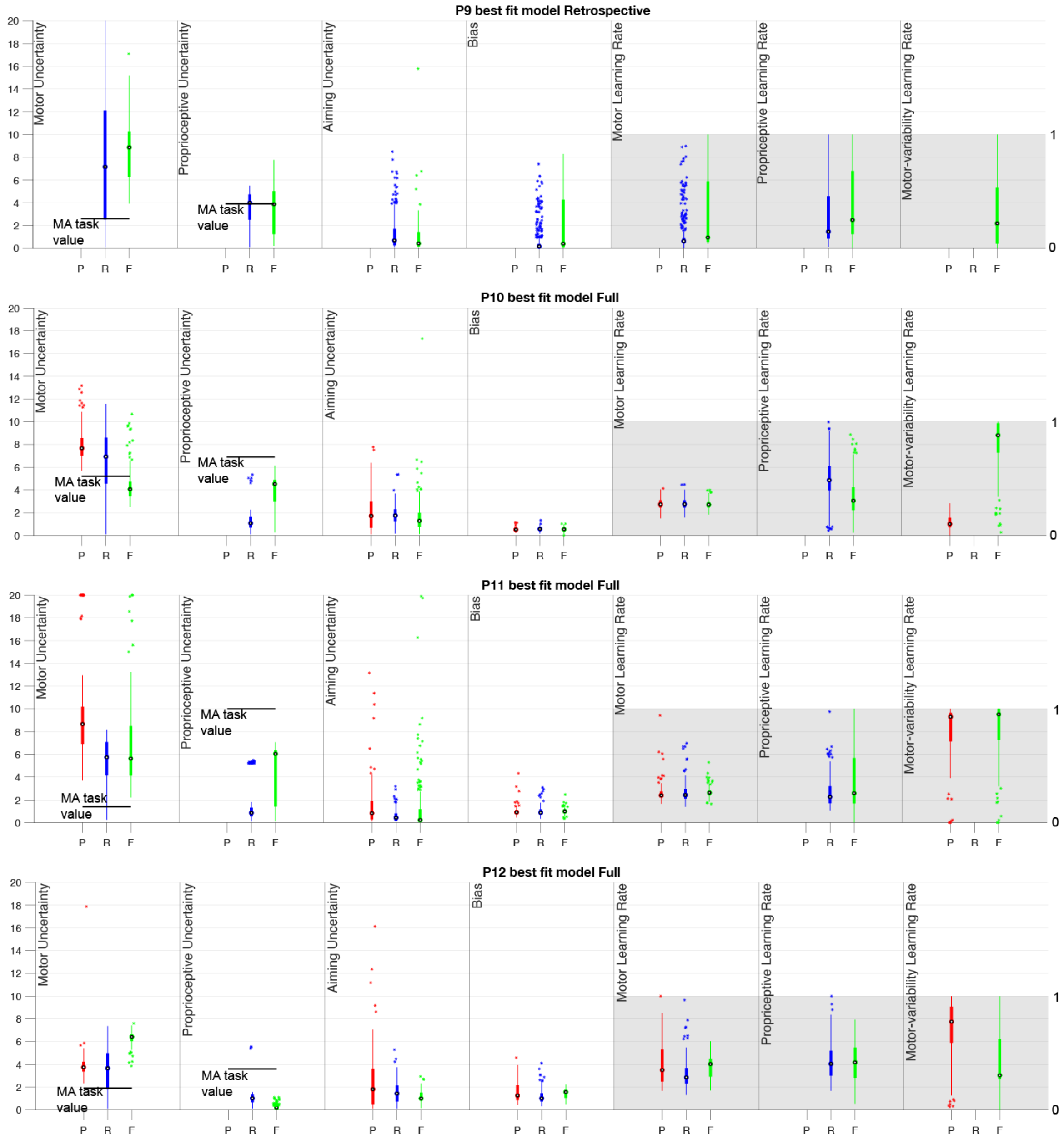

bioRxiv preprint doi: https://doi.org/10.1101/2025.10.31.685783; this version posted November 1, 2025. The copyright holder for this preprint (which was not certified by peer review) is the author/funder, who has granted bioRxiv a license to display the preprint in perpetuity. It is made available under aCC-BY 4.0 International license.

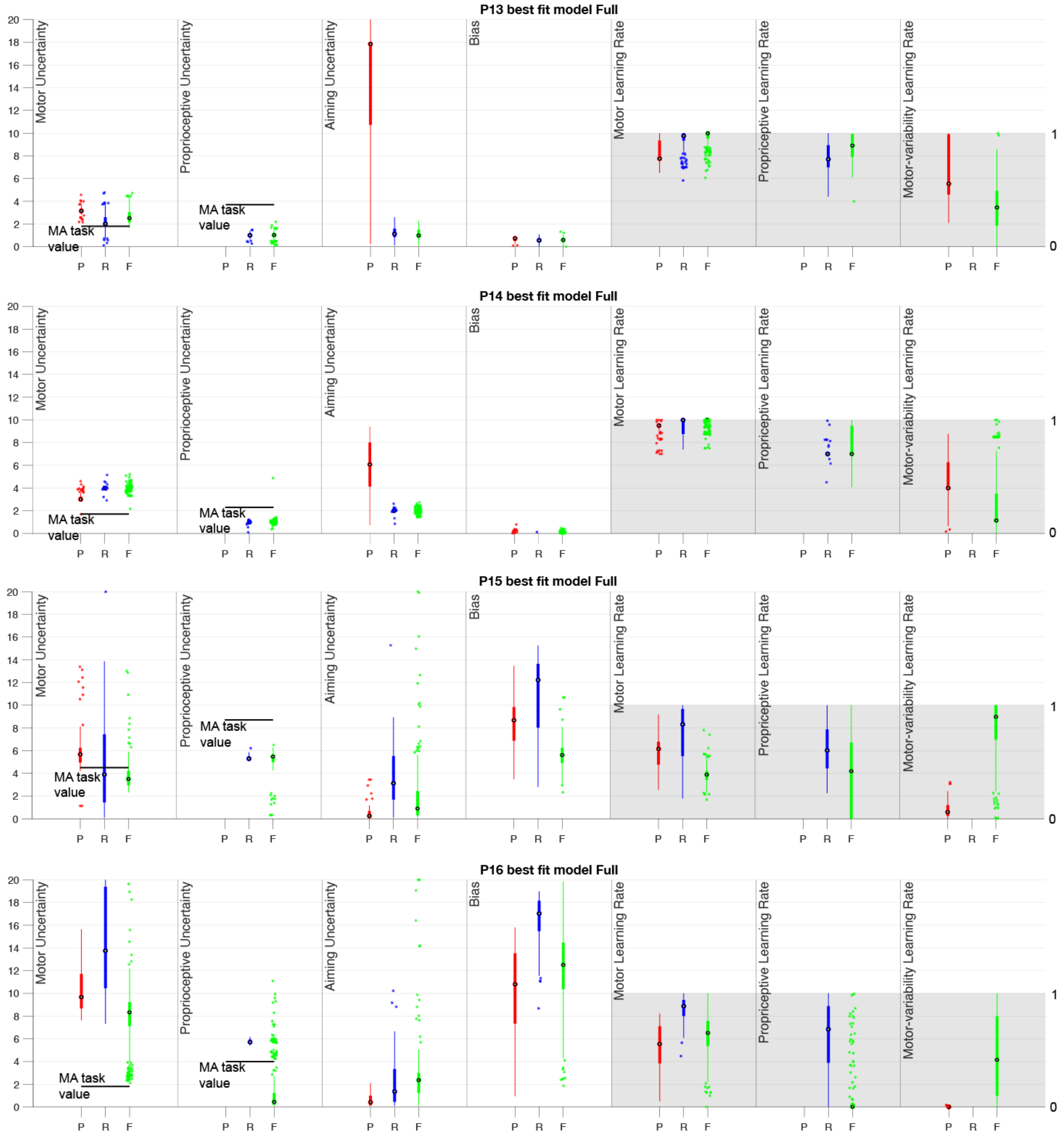

bioRxiv preprint doi: https://doi.org/10.1101/2025.10.31.685783; this version posted November 1, 2025. The copyright holder for this preprint (which was not certified by peer review) is the author/funder, who has granted bioRxiv a license to display the preprint in perpetuity. It is made available under aCC-BY 4.0 International license.

Supplement 2: Parameter comparisons for each model fit.
For each participant we've plotted the range of parameters resulting in a winning iteration of a given model. On the *x*-axis the three models are represented for each parameter, Prospective (P) in red, Retrospective (R) in blue, and Full (F) in green. There are 12 sets of parameters for each iteration so there are between 24 and 444 data points for each model, depending on the number of winning iterations. These box plots show the parameters from the fits that resulted in the winning model producing the lowest least-squares value and winning that round. If the model did not use a particular parameter it has been left blank here. If that participant never had a winning iteration of a given model, there are no winning parameters to show and the model's section has been left blank. The box edges are the upper and lower quartiles, with the horizontal line reflecting the median, dots representing outliers (calculated using the interquartile range) and whiskers reflecting maximum and minimum values that are not outliers. The black bars in the motor- and proprioceptive-error plots are the values fit for each participant in the separate motor-awareness task. None of the data used to generate those values was used in calculating the parameters shown here and vice versa.
